# Strategies to implement and monitor in-home transcranial electrical stimulation in neurological and psychiatric patient populations: a systematic review

**DOI:** 10.1186/s12984-019-0529-5

**Published:** 2019-05-15

**Authors:** Nandini Sandran, Susan Hillier, Brenton Hordacre

**Affiliations:** 1 Body in Mind, Division of Health Sciences, University of South Australia, City East Campus, GPO Box 2471, Adelaide, 5001 South Australia; 2 Division of Health Sciences, University of South Australia, Adelaide, South Australia

**Keywords:** Non-invasive brain stimulation, Transcranial direct current stimulation, Rehabilitation, Home therapy, Remote monitoring, Telemedicine

## Abstract

**Background:**

Transcranial electrical stimulation is a promising technique to facilitate behavioural improvements in neurological and psychiatric populations. Recently there has been interest in remote delivery of stimulation within a participant’s home.

**Objective:**

The purpose of this review is to identify strategies employed to implement and monitor in-home stimulation and identify whether these approaches are associated with protocol adherence, adverse events and patient perspectives.

**Methods:**

MEDLINE, Embase Classic + Embase, Emcare and PsycINFO databases and clinical trial registries were searched to identify studies which reported primary data for any type of transcranial electrical stimulation applied as a home-based treatment.

**Results:**

Nineteen published studies from unique trials and ten on-going trials were included. For published data, internal validity was assessed with the Cochrane risk of bias assessment tool with most studies exhibiting a high level of bias possibly reflecting the preliminary nature of current work. Several different strategies were employed to prepare the participant, deliver and monitor the in-home transcranial electrical stimulation. The use of real time videoconferencing to monitor in-home transcranial electrical stimulation appeared to be associated with higher levels of compliance with the stimulation protocol and greater participant satisfaction. There were no severe adverse events associated with in-home stimulation.

**Conclusions:**

Delivery of transcranial electrical stimulation within a person’s home offers many potential benefits and appears acceptable and safe provided appropriate preparation and monitoring is provided. Future in-home transcranial electrical stimulation studies should use real-time videoconferencing as one of the approaches to facilitate delivery of this potentially beneficial treatment.

## Introduction

Transcranial electrical stimulation (tES) is a technique used to modulate cortical function and human behaviour. It involves weak current passing through the scalp via surface electrodes to stimulate the underlying brain. A common type of tES is transcranial direct current stimulation (tDCS). Several studies have demonstrated tDCS is capable of modulating cortical function, depending on the direction of current flow [1–3]. When the anode is positioned over a cortical region, the current causes depolarisation of the neuronal cells, increasing spontaneous firing rates [4]. Conversely, positioning the cathode over the target cortical region causes hyperpolarisation and a decrease in spontaneous firing rates [4]. This modulation of cortical activity can be observed beyond the period of stimulation and is thought to be mediated by mechanisms which resemble long term potentiation and depression [5]. Along similar lines, transcranial alternating current stimulation (tACS) and transcranial random noise stimulation (tRNS) are also forms of tES. Both tACS and tRNS are thought to interact with ongoing oscillatory cortical rhythms in a frequency dependent manner to influence human behaviour [6–8].

The ability of tES to selectively modulate cortical activity offers a promising tool to induce behavioural change. Indeed, several studies have demonstrated that tES may be a favourable approach to reduce impairment following stroke [9], improve symptoms of neglect [10], or reduce symptoms of depression [11]. While these results appear promising, there remains debate around technical aspects of stimulation along with individual participant characteristics that may influence the reliability of a stimulation response [12–22]. However, current evidence does suggest that effects of stimulation may be cumulative, with greater behavioural improvements observed following repeated stimulation sessions [20]. Furthermore, tES has shown potential as a tool for maintenance stimulation, with potential relapses of depression managed by stimulation which continued over several months [23, 24]. Therefore, it may be that repeated stimulation sessions will become a hallmark of future clinical and research trials aiming to improve behavioural outcomes. This would require participants to attend frequent treatment sessions applied over a number of days, months or years. Given that many participants who are likely to benefit from stimulation are those with higher levels of motor or cognitive impairment, the requirement to travel regularly for treatment may present a barrier, limiting potential clinical utility or ability to recruit suitable research participants [25]. In addition, regular daily treatments would also hinder those who travel from remote destinations to receive this potentially beneficial neuromodulation. Therefore, there is a requirement to consider approaches to safely and effectively deliver stimulation away from the traditional locations of research departments or clinical facilities.

One benefit of tES over other forms of non-invasive brain stimulation, such as repetitive transcranial magnetic stimulation, is the ability to easily transport the required equipment. This opportunity may allow for stimulation to be delivered in a participant’s home, which could represent the mode of delivery for future clinical applications. However, it may be unreasonable to expect that a participant would be capable of managing delivery of tES alone and would likely require some form of training and/or monitoring [25]. Although tES is considered relatively safe [26], stimulation should be delivered within established guidelines to avoid adverse events [27]. Inappropriate delivery of stimulation could result in neural damage, detrimental behavioural effects, irritation, burns or lesions of the skin [28–33]. Therefore, in order to deliver stimulation safely to the appropriate cortical region, it is likely that in-home stimulation may require some form of monitoring [25].

It is currently unclear what the best approach is to implement and monitor in-home tES. An early paper proposed several guidelines to perform in home tES [34]. However, these guidelines were not based on evidence from published clinical trials as there were none available at the time of publication. One recent systematic review sought to discuss current work in this area and highlighted the need for further research to investigate safety, technical monitoring and assessment of efficacy [35]. Given the recent, and growing, interest in home-based brain stimulation, we felt it was now pertinent to conduct a review to specifically identify strategies employed to implement and monitor the use of in-home tES in neurological and psychiatric populations. The secondary questions were to report protocol adherence, adverse events and patient perspectives of in-home tES. Understanding optimal treatment fidelity for in-home brain stimulation will be instrumental to achieving higher levels of tES useability and acceptance within a participant’s home.

## Methods

**Fig. 1:**
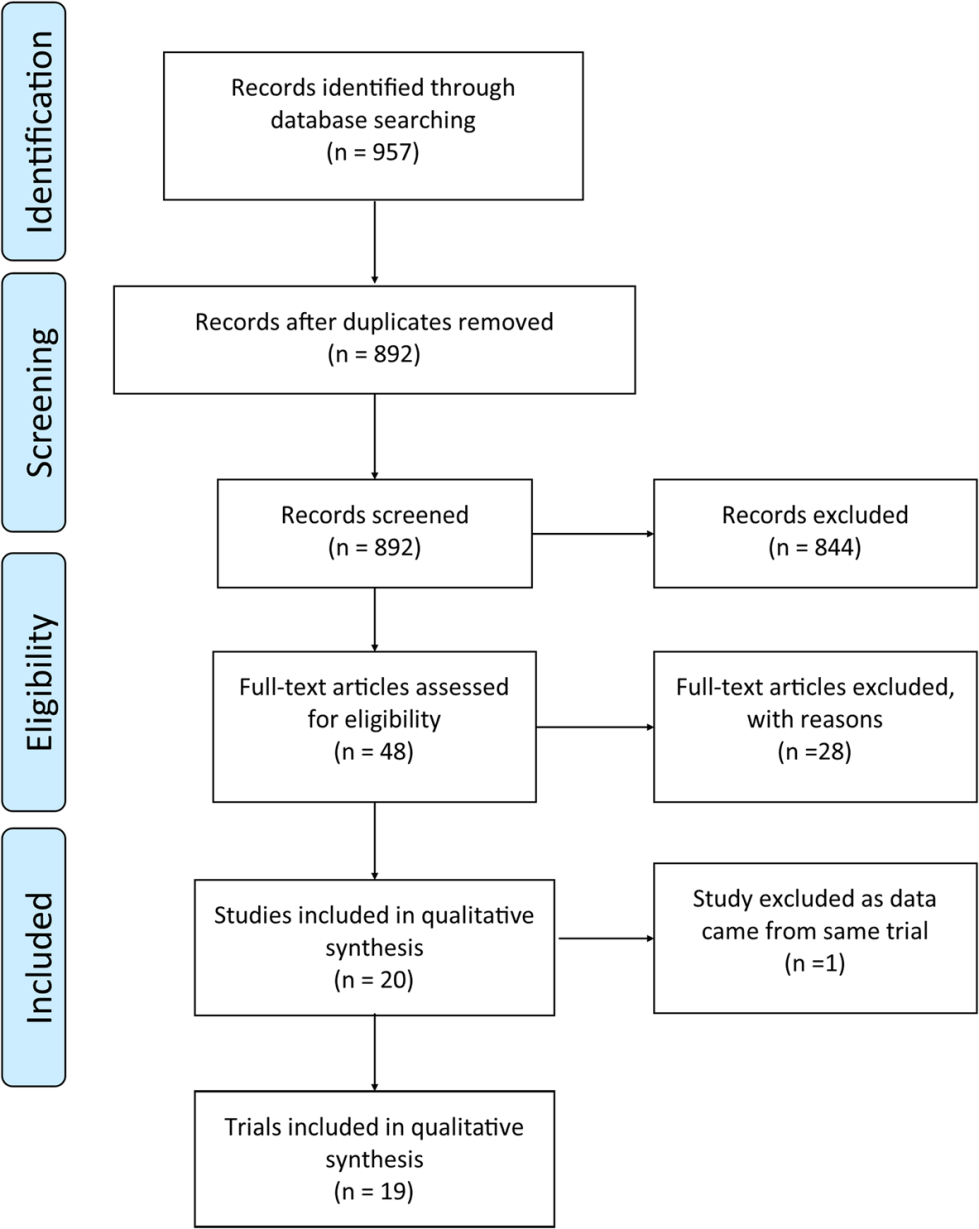
PRISMA flow diagram of study inclusion for this systematic review

This systematic review adhered to the Preferred Reporting Items for Systematic Reviews and Meta-Analysis (PRISMA) guidelines (Fig. 1) and is registered with the International Prospective Register of Systematic Reviews (PROSPERO Registration number CRD42018091960). The literature search of studies that involve in-home tES was performed in the following databases; MEDLINE (1946 to February 2019), Embase Classic + Embase (1947 to February 2019), Emcare (1995 to February 2019) and PsycINFO (1806 to February 2019). In addition, trial registries (clinicaltrials.gov, anzctr.org.au and who.int/ictrp/en/) were searched to identify current in-home tES studies. The search was carried out in February 2019 using combinations of keywords relating to in-home tES (search strategy available on PROSPERO, CRD42018091960).

Studies were included if (i) they presented primary data using any type of tES including tDCS, tRNS or tACS as the treatment approach regardless of types of montage as well as whether it was applied with or without additional therapy (ii) the tES treatment was carried out as a home-based treatment (iii) the study involved humans of any age with neurological or psychiatric conditions which included, but was not limited to, stroke, Parkinson’s disease, traumatic brain injury and depression (iv) the study reported on compliance with treatment protocol, adverse events or participant satisfaction with home stimulation (v) the type of study was either a randomised controlled trial, randomised cross-over trial, observational study, case series, or qualitative study, and (vi) the study was published or available in the English language. All other studies that did not fulfil the inclusion criteria were excluded.

The review process was carried out in three steps. First, one reviewer (N.S.) screened titles and abstracts according to eligibility criteria and excluded studies which were obviously not related to the search criteria. Second, full text articles were retrieved and screened by two reviewers (B.H. and N.S.) with a third reviewer (S.H.) resolving any discrepancies that arose. The reference lists of included articles were then screened to identify any additional articles that may not have been found during the original search. Third, quality assessment of each included study was carried out using the Cochrane risk of bias assessment [36]. Two reviewers (B.H. and N.S.) assessed bias individually and discrepancies were resolved via discussion. The variables extracted from the included papers were; (1) subject demographics and clinical characteristics (age, gender, sample size, clinical condition(s)), (2) details on strategies to facilitate in-home tES treatment, (3) type of stimulator used, (4) size and positioning of electrodes, (5) parameters of stimulations for in-home sessions (current and duration), (6) monitoring approaches, (7) adverse events, (8) study protocol compliance, which was defined as the percentage of correctly completed stimulation sessions relative to the total number of intended sessions, and (9) perception of patients towards in-home tES treatment. Where required, authors were contacted to obtain additional data. Data were then synthesised using a narrative approach to describe approaches to achieve optimal treatment fidelity. This included strategies employed both prior to, and during, the treatment period to effectively implement in-home tES and approaches to monitor use of in-home tES during the treatment period to ensure it is both effective and safe. Where reported, adverse events, protocol adherence and participant perspectives were also synthesised using a narrative approach and discussed with reference to approaches to implement and monitor in-home tES.

## Results

### Study description

A total of 957 related studies were identified, with 65 duplicates removed, leaving 892 studies for title and abstract screening. Following title and abstract screening, 48 studies remained and full text articles of those studies were screened for eligibility. Out of these 48 studies, 28 were discarded as 11 studies were found to have the wrong study design (three review articles, one book chapter, six methodological or guideline papers and one protocol paper) and one had the wrong study population. The other 16 studies were considered duplicates as they were conference abstracts related to full text studies already included in the review. Thus, in total, this systematic review included 20 studies. One additional study [37] was excluded from this review as it was identified through communication with the author that the data came from a trial that was already included within the review [38]. As B Dobbs, N Pawlak, M Biagioni, S Agarwal, M Shaw, G Pilloni, M Bikson, A Datta and L Charvet [38] reported all outcomes of interest to this review, it was decided that data would be extracted from this study. As a result, there were 19 studies identified with data from unique trials (Fig. 1).

### Description of included studies

All 19 studies are summarised in Table 1. Although the inclusion criteria for this review included several variations of tES, all identified studies used tDCS as the form of brain stimulation, with none using tACS or tRNS. The studies were relatively recent, all being published within the past 5 years (2013–2018), while 84% were published within the last 2 years (2016–2018). Of the included studies, five were identified as randomised controlled trials [39–43], two randomised cross-over trials [44, 45], two observational studies [46, 47], ten case series [38, 48–56].

**Table 1:** Descriptions of studies based on sample populations and stimulation parameters

Study	Study Design and Population					Stimulation Parameters					
	Design	Size	Gender	Age (years)	Condition	Type	Target Area	Electrodes	Current	Session	Duration
Andrade 2013	Case series	1	1F	25	Schizophrenia	Zeebeetronics	F3 (Anodal tDCS)	25cm^2^	1–3 mA	3 years	20–30 min
Andre 2016	RCT	22	NS	63–94	Dementia	NeuroConn	DLPFC (Anodal tDCS)	35cm^2^	2 mA	4 sessions	20 min
Carvalho 2018	Case series	1	1F	57	Neuropathic pain	Neuroelectric Starstim	M1 (Anodal tDCS)	35cm^2^	2 mA	5 sessions	20 min
Cha 2016	RCT	24	24F	52.9 ± 12.2	MdDS	Transcranial Technologies	DLPFC	35cm^2^	1 mA	20 sessions	20 min
Charvet 2017a	Observational	25	21F, 4 M	51.0 ± 12.7	MS	Soterix Mini-Clinical Trials	DLPFC (Anodal tDCS)	35 cm2	1.5 mA	10 sessions	20 min
Charvet 2017b^1^	RCT	27	16F, 11 M	44.2	MS	Soterix Mini-Clinical Trials	DLPFC (Anodal tDCS)	25 cm2	2 mA	20 sessions	20 min
Clayton 2018	Case series	1	1F	54	MS, Bipolar	NS	DLPFC (Anodal tDCS)	NS	2 mA	40 sessions	20 min
Dobbs 2018	Case series	16	4F, 12 M	66.9 ± 5.4	PD	Soterix Mini-Clinical Trials	DLPFC (Anodal tDCS)	25cm^2^	2 mA	10 sessions	20 min
Hagenacker 2014	RCT - crossover	17	10F, 7 M	32–82	Trigeminal neuralgia	Neuronica	M1 (Anodal tDCS)	16cm^2^	1 mA	14 sessions	20 min
Hyvarinen 2016	Observational	43	20F, 23 M	51 ± 15.4	Tinnitus	Sooma	Auditory or bifrontal tDCS	35cm^2^	2 mA	10 sessions	20 min
Kasschau 2016	Case series	20	17F, 3 M	30–69	MS	tDCS mini-Clinical Trial	DLPFC	25cm^2^	1.5 mA	10 sessions	20 min
Loo 2017	Case series	10	NS	NS	Depression	NS	DLPFC	NS	2 mA	20 sessions	30 min
Martens 2018	RCT - crossover	27	8F,19M	42 ± 14.5	MCS	CEFALY tDCS	DLPFC	35cm^2^	2 mA	20 sessions	20 min
Mortenson 2016	RCT	16	7F, 9 M	44–76	Stroke	NeuroConn	M1 (Anodal tDCS)	35cm^2^	1.5 mA	5 sessions	20 min
Riggs 2018	Case series	4	2F, 2 M	44–63	Multiple conditions	Soterix Mini-Clinical Trials	DLPFC/M1 (Anodal tDCS)	25cm^2^	1–1.5 mA	10 sessions	20 min
Schwippel 2017	Case series	1	1 M	31	Hallucinations	Neuroconn	Tempoproparietal (Anodal tDCS)	35cm^2^	2 mA	400 sessions	20 min
Sharma 2018	RCT	12	NS	64.7 ± 7.1	PD	NS	DLPFC	NS	2 mA	10 sessions	20 min
Treister 2015	Case series	11	7F, 4 M	60.6 ± 16.3	Neurological pain	NS	M1 (Anodal tDCS)	NS	1.5 mA	12 sessions	20 min
Van de Winckel 2018	Case series	6	3F, 3 M	61 ± 10	Stroke	StartSim	M1 bihemispheric	20cm^2^	1.5 mA	5 sessions	20 min

DLPFC = Dorsolateral prefrontal cortex; F = female; M = male; MCS = Minimally conscious state; MdDS = Mal De Debarquement Syndrome; MS = Multiple sclerosis; M1 = primary motor cortex; NS = not stated; PD = Parkinson’s disease; RCT = Randomised controlled trial; tDCS = transcranial Direct Current Stimulation

^1^, data only reported for study two

### Risk of bias in included studies

**Fig. 2:**
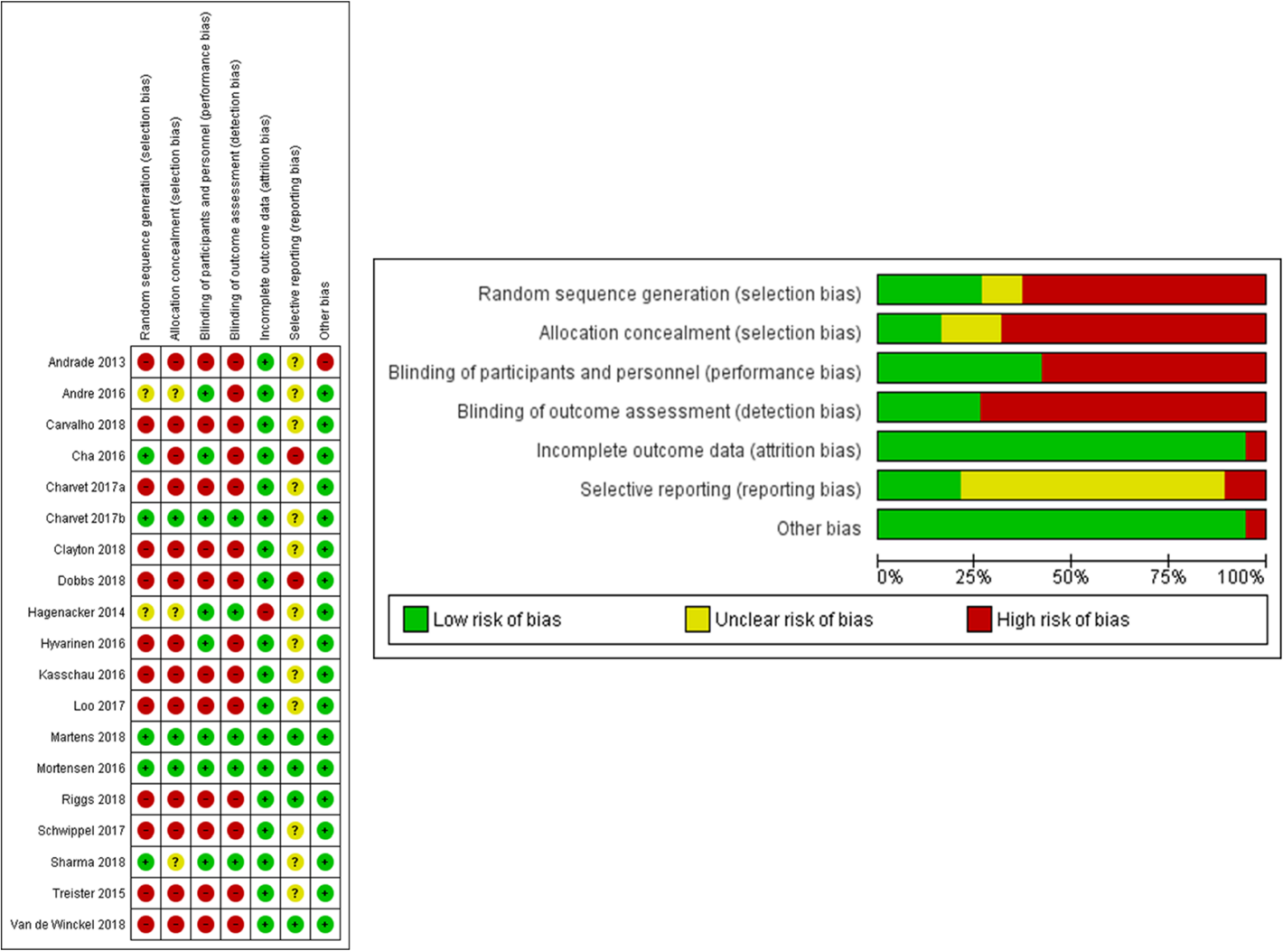
Cochrane risk of bias tool was used to assess quality of included studies

Review of internal validity using the Cochrane risk of bias assessment tool in seven domains is summarised in Fig. 2. In keeping with the low level of research design, which likely reflects the preliminary nature of current work, the majority of studies had a high risk of bias. Few studies (26.3%) demonstrated low risk for random sequence generation [39–41, 43, 45], allocation concealment (15.8%) [40, 41, 45], blinding of personnel and participants (42.1%) [39–46] and blinding of outcome assessments (26.3%) [40, 41, 43–45]. However, a large proportion of studies (94.7%) demonstrated low risk of bias for incomplete outcome data, with only one study identified as high risk of bias due to an increased number of participants who withdrew from the study [44]. Selective reporting was generally (68.4%) identified as an unclear level of bias [40, 42–44, 46–49, 51–55], with two studies identified as high risk of bias [38, 39], and one as low risk of bias [41] in this category. One study was identified as high risk of bias under the domain of other sources of bias due to substantial variation in duration and intensity of stimulation based on response to treatment [52]. However, we note that the overall purpose of this study was different compared to all other included studies as it was a maintenance program for symptoms of schizophrenia.

### Participants’ characteristics and stimulation protocols

There were various patient populations included in this review for in-home tES (Table 1). Four studies were performed with people who had Multiple Sclerosis (MS) [40, 47, 48, 54], two with Parkinson’s disease (PD) [38, 43] and two with stroke [41, 56]. Other populations included tinnitus [46], dementia [42], minimally conscious state [45], Mal de Debarquement syndrome [39], trigeminal neuralgia [44], neuropathic pain [55], depression [49], multimodal hallucinatory perceptions [53], schizophrenia [52], various neurological pain conditions [51] and a case series of four chronically ill patients which included myasthenia gravis, depression, chronic pain and stroke [50].

In-home tES treatment was provided over a wide range of different durations from 4 [42] to 400 sessions [53], with the most common approach to apply in-home tES for 10–20 sessions [38–40, 43–51]. One study did not report the number of treatment sessions which ranged from once to twice daily over a period of 3 years [52]. The duration for each treatment was 20–30 min for all included studies, with the majority applying stimulation at 1-2 mA. Only one study exceeded 2 mA, with stimulation intensity increased up to 3 mA to control symptoms of Schizophrenia [52].

### Approaches to achieve optimal treatment fidelity for in-home brain stimulation

Across the 19 included studies, there were a range of strategies used both prior to, and during, the treatment period to implement in-home tES (Table 2). The most common approach was to conduct training sessions prior to beginning in-home tES. This frequently included practicing the placement and positioning of electrodes on the scalp, sponge preparation, starting the stimulator, troubleshooting common problems and provision of training videos [39, 46, 53]. Furthermore, several studies extended the training sessions to include a caregiver, or support person, who was able to assist during the home treatment phase. For some studies, the assistance of a caregiver, or support person, was a requirement for all participants [44, 48, 50, 55], and others specifying it only for those participants with higher disability [38, 40, 47].

**Table 2:** Strategies identified to implement in-home tES

Study	Strategies to implement in-home tES					
	Training session	Caregiver training	Customised headband	Remote computer access (stimulator set-up)	Home visit	Stimulation delivered by research team
Kasschau 2016	✓	✓	✓	✓	✓	
Riggs 2018	✓	✓	✓	✓	✓	
Dobbs 2018	✓	✓	✓	✓		
Sharma 2018	✓	✓	✓	✓		
Van de Winckel 2018	✓	✓	✓	✓		
Charvet 2017a	✓	✓	✓	✓		
Charvet 2017b	✓	✓	✓	?		
Carvalho 2018	✓	✓				
Hagenacker 2014	✓	✓				
Cha 2016	✓		✓			
Hyvarinen 2016	✓		✓			
Schwippel 2017	✓					
Treister 2015	✓					
Loo 2017	✓					
Mortenson 2016						✓
Martens 2018		✓				
Andrade 2013						
Andre 2016						
Clayton 2018						

*Note; Andrade 2013, Andre 2016 and Clayton 2018 did not report any strategy to implement in-home tES*

✓; Reported as a strategy to implement in-home tES

*?; Charvet 2017b reported each participant was provided a laptop that enabled connection to a study technician. It is not stated whether this included remote computer access*

During delivery of in-home tES, several monitoring approaches were identified as strategies to achieve optimal treatment fidelity (Table 3). The most common treatment monitoring approach was to use videoconferencing to observe, in real time, the in-home treatment being conducted by the patients or their caregivers [38–40, 43, 47–50, 54–56]. This provided researchers opportunity to visualise tES set-up, correct electrode placement and troubleshoot issues that arose. Monitoring in-home stimulation in this manner may also assist with compliance with the treatment protocol. Five studies which utilised videoconferencing as a method to monitor in-home tES also used a remote desktop access approach for each treatment [38, 43, 47, 48, 50, 56]. This allowed research staff to have strict dose control for the delivery of tES and remotely solve any technology-based issues that arose. Four studies used passive (not in real time) monitoring approaches which included recording use of tES through websites such as Survey Monkey [39] or with self-report treatment diaries [44–46].

**Table 3:** Monitoring approaches and protocol compliance of the studies

Study	Monitoring approaches	Compliance
Mortenson 2016	Direct in-person monitoring	100%
Clayton 2018	Real-time videoconferencing	100%
Carvalho 2018	Real-time videoconferencing	100%
Van de Winckel 2018	Real-time videoconferencing Remote administration of tDCS delivery	100%
Riggs 2018	Real-time videoconferencing Remote administration of tDCS delivery	100%
Dobbs 2018	Real-time videoconferencing Remote administration of tDCS delivery	100%
Sharma 2018	Real-time videoconferencing Remote administration of tDCS delivery	100%
Charvet 2017a	Real-time videoconferencing Remote administration of tDCS delivery	> 96%
Cha 2016	Real-time videoconferencing Daily online-tracking and reporting system	96%
Kasschau 2016	Real-time videoconferencing Remote administration of tDCS delivery	96%
Charvet 2017b^1^	Real-time videoconferencing Remote administration of tDCS delivery	93%
Hyvarinen 2016	Daily self-report treatment diary	91%
Loo 2017	Initial sessions real-time videoconferencing Daily phone or email contact	90%
Martens 2018	Daily self-report abnormalities, questionnaire	81%
Hagenacker 2014	Daily self-report treatment diary	76%

^1^, data only reported for study two

### Protocol compliance

Table 3 shows that most studies reported a high level of compliance with the in-home tES treatment program which was defined as the percentage of correctly completed stimulation sessions relative to the total number of intended sessions. Seven studies had 100% compliance, with three additional studies having 95% compliance or greater and a further three studies reporting 90–95% compliance. These results suggest that it is possible to implement an in-home tES study and obtain an excellent level of compliance. An observation from this review is that studies which provided regular and repeated real-time videoconferencing to monitor each in-home treatment session achieved compliance levels of 93% or greater [38–40, 43, 47, 48, 50, 54–56]. One study delivered in-home tES with research staff attending a participant’s home as opposed to the study participant performing stimulation independently, achieving 100% compliance [41].

The studies which did not use real-time videoconferencing to monitor each in-home treatment session reported different approaches that achieved comparatively lower compliance levels (see Table 3). These strategies included a single real-time videoconference call for the first in-home treatment session only, daily phone calls and emails and self-reported treatment diaries [44–46, 49]. Although there may be some indication that strategies to monitor in-home tES may influence protocol compliance, there are likely to be additional factors which contribute to this outcome. For example, it is important to acknowledge that protocol compliance may be affected by the duration of the experiment with high levels of compliance likely to be more difficult to achieve with more home stimulation sessions. Within this review, those studies with relatively higher levels of protocol compliance generally conducted 5–20 sessions, while those with relatively lower levels of compliance conduced 10–20 sessions.

As opposed to monitoring strategies, where videoconferencing appears to be a factor that may enable high levels of protocol compliance, there did not appear to be any association between a particular strategy to implement in-home tES and protocol compliance (Table 2). For example, some of the studies which reported relatively high levels of compliance used various strategies to implement in-home tES such as training sessions for participants and caregivers [38, 43, 47, 48, 50, 55, 56], customised headbands [38–40, 43, 47, 48, 50, 56] and remote computer access [38, 43, 47, 48, 50, 56], while others did not report any strategies [54]. However, it may be that use of multiple strategies is best. Of the studies which used three or more strategies to prepare the participant and deliver the in-home tES program, reported compliance levels were between 93 and 100% [38, 40, 43, 47, 48, 50, 56]. For studies which used two strategies or less, compliance levels appeared more variable and were as low as 76% [44].

### Adverse events

Eighteen studies reported outcomes for adverse events (Table 4). The most common event was tingling sensations during the stimulation, which was reported in 12 studies [38–41, 43, 44, 46, 48, 52, 54–56]. Other common adverse events were itching or skin irritation, burning sensation, head pain, difficulties in concentrating, blurred vision, facial muscle twitching and changes in mood. There did not appear to be any association between adverse events and strategies to implement or monitor in-home tES. One study reported occurrence of a seizure, however the authors suggest this was not associated with stimulation as the participant was receiving a sham condition (no stimulation) and had a history of epilepsy [45]. Excluding the occurrence of this seizure which did not appear to be associated with delivery of tDCS, none of the reported adverse events would be considered severe or requiring medical attention.

**Table 4:** Reported adverse events for in-home tES

	Adverse Events
Andrade 2013	Tingling sensation at the electrode site
Andre 2016	No adverse events occurred
Carvalho 2018	Minor and transient scalp burn, tingling and skin redness
Cha 2016	Tingling, itching, redness, headache, tiredness, confusion, nausea.
Charvet 2017a	Not stated
Charvet 2017b^1^	Pain > 6/10 (*n* = 2). Tingling (43%), itching (21%), burning sensation (23%), head pain or pressure (2%), dizziness (< 1%), difficulty concentrating (4%), blurred vision (<1%) and facial muscle twitch (<1%).
Clayton 2018	Minimal and transient tingling and itchiness at the site of electrodes
Dobbs 2018	Tingling (43%), burning (29%), head pain (8%), itching (8%), headache (6%), difficulty concentrating (1%)
Hagenacker 2014	Slight itching or tingling
Hyvarinen 2016	Tinnitus loudness and annoyance (5%), skin burn (2%); irritation (2%), mood changes (2%), tingling sensation (5%), uncomfortable sensation (47%), poor sleep (7%)
Kasschau 2016	Tingling (60%), itching (24%), burning (30%), headache (3%), nausea (3%), head pain (3%), dizziness (<1%), difficulty concentrating (1%), blurred vision (<1%), forgetfulness (<1%).
Loo 2017	No significant adverse events occurred
Martens 2018	Skin redness (37%), sleepiness (11%), seizure (4%)*
Mortenson 2016	Mild itching, tingling, burning sensation, headache and sleepiness
Riggs 2018	No adverse events occurred
Schwippel 2017	No adverse events occurred
Sharma 2018	Tingling (20%), itching (6.5%), burning (8.3%), dizziness (0.4%), headache (2.2%), sleepiness (0.4%, nausea (0.4%)
Treister 2015	No serious adverse events occurred
Van de Winckel 2018	Mild tingling at electrode site at beginning of treatment (83%)

^1^, data only reported for study two

*Unlikely related to stimulation as it occurred in the sham group in a participant with history of epilepsy

### Participants’ satisfaction

Participants’ satisfaction towards the treatment was only reported by six studies [39, 46, 48, 50, 55, 56]. For five studies where real-time monitoring was provided, but a range of different strategies were employed to prepare participants for home stimulation, authors reported that participants generally had positive experiences with using in-home tES [39, 48, 50, 55, 56]. These studies were performed in various clinical populations that included neuropathic pain, Mal de Debarquement Syndrome, stroke, depression, myasthenia gravis, chronic pain and Multiple Sclerosis. Themes that emerged included users reporting that there were no difficulties in the treatment set-up, being comfortable with the stimulation device, being satisfied with the overall experience and expressing a desire to continue this home-based treatment after the study [48, 50, 55, 56] with purchase of their own stimulator [39]. However, some participants with Mal de Debarquement Syndrome, a condition characterised by feelings of rocking or swaying after exposure to motion, reported feeling uncomfortable applying the stimulation independently and reported frustration setting up the device and achieving appropriate levels of impedance to start stimulation [39]. The fourth study reported the perspectives of people with tinnitus who used in-home tES and were provided with self-reported treatment diaries to monitor stimulation [46]. The authors reported six out of 35 participants felt it was difficult to apply stimulation despite being providing with a one-day training session and instruction notes.

### Registered clinical trials

The search of trial registries (clinicaltrials.gov, anzctr.org.au and who.int/ictrp/en/) identified ten clinical studies which are currently ongoing or completed, but not yet published (Table 5). These studies implemented in-home tES in various neurological and psychiatric conditions. Four studies stated that videoconferencing (or telemedicine) will form part of the monitoring strategy to implement in-home tES. Few studies have identified that they will be reporting on the occurrence of adverse events, protocol compliance or patients’ perspective. However, it is worth noting that limited information is required to be provided in clinical trial registries.

**Table 5:** Clinical trials which are currently investigating in-home tES in neurological or psychiatric conditions

Registration No.	Status	Population	Intervention	Strategies to implement or monitor in-home tES	Results to be reported		
					Adverse events	Compliance	Patients’ perspective
NCT03499314	Recruiting	Multiple Sclerosis	Anodal tDCS and dexterity training (20 sessions)	Real-time videoconferencing	?	?	?
NCT03189472	Recruiting	Parkinson’s Disease	tDCS (5 weeks)	Real-time videoconferencing Individual ‘unlock’ codes for stimulation Remote computer access	?	?	?
NCT02959502	Recruiting	Alzheimer’s Dementia	tDCS and cognitive training (40 sessions, 8 weeks)	Caregiver will be trained in tDCS. No monitoring strategy reported	?	?	?
NCT02894736	Recruiting	Major Depression	tDCS	Monitoring strategy not reported	?	?	?
NCT02746705	Recruiting	Multiple Sclerosis	tDCS and cognitive training (20 sessions, 4 weeks)	Real-time videoconferencing	?	?	?
NCT02652988	Recruiting	Fibromyalgia	tDCS (60 sessions, 12 weeks)	Not reported	?	?	?
NCT02346396	Recruiting	Neuropathic chronic pain	tDCS (20 sessions, 4 weeks)	Monitoring strategy not reported	?	?	?
ISRCTN56839387	Completed	Chronic Pain	tDCS (5 sessions)	Not reported	?	?	?
ACTRN12618000443291	Recruiting	Stroke	tDCS and arm exercise (14 sessions, 2 weeks)	Real-time videoconferencing	?	✓	?
ACTRN12615000592549	Recruiting	Tourette Syndrome	tDCS (18 sessions, 9 weeks)	Remotely supervised (strategy not stated)	?	?	?

*tDCS* transcranial direct current stimulation

Tick = reported in trial registry

? = not clearly reported in trial registry

## Discussion

It is clear that in-home tES is an area of current research interest. Of the studies identified in this review, the majority were published within the last few years. Generally, these studies have been conducted in relatively small samples of patients, suggesting that current work is mostly around testing feasibility, safety and tolerability of this approach. As a result, many studies were found to have high levels of bias due to the less rigorous research designs employed. Nevertheless, this review identified a number of key findings from current evidence which will inform future in-home tES research and clinical practice. First, several different strategies were identified to implement and monitor in-home tES. The requirement to monitor stimulation appears to be a critical component to the successful implementation of in-home tES. Second, studies that employ real-time videoconferencing as a strategy to monitor in-home tES seemed to be associated with increased compliance with stimulation protocols and could possibly contribute to greater patient satisfaction with this form of treatment. Finally, from the available evidence, there does not appear to be any indication of severe adverse events associated with stimulation, suggesting that in-home tES is safe. Considering these findings in light of the promising advantages of delivering tES within a participant’s home, we suggest future studies continue to explore this approach.

The primary aim of this review was to identify approaches to implement and monitor in-home tES and achieve optimal treatment fidelity. We were particularly motivated to investigate this research question because in-home tES is likely to require significant consideration to ensure stimulation is performed correctly and in accordance with the treatment program, while maintaining patient safety. Therefore, strategies to implement and monitor in-home tES will be essential to ensure correct set-up and electrode preparation prior to stimulation, as well as to troubleshoot any issues that arise. It is clear from the available evidence that several approaches have been employed and these are likely to be critical aspects of future work for in-home tES. It is of particular note that studies which used real-time videoconferencing to monitor stimulation remotely were associated with higher levels of compliance (93–100%) with the in-home tES program [38–40, 43, 47, 48, 50, 54–56]. These are impressive levels of protocol compliance that are comparable to, or even greater, than that reported in previous clinical trials or research studies [9, 57, 58]. It should be noted that several of these studies did follow similar methodology as they followed an earlier guidelines paper that proposed a comprehensive approach to implement in-home tES [34]. However, despite similarity in their methodology, it is interesting to note that higher levels of compliance were observed across several different clinical populations and unique datasets. It is perhaps not surprising this approach resulted in greater compliance with the treatment protocol. Regular contact and viewing each treatment session in real-time likely ensured that technical or set-up difficulties were appropriately dealt with in an efficient and timely manner, preventing these challenges from affecting treatment compliance. There is also likely to be opportunity for research staff to motivate and encourage participants to continue with the treatment protocol where regular videoconferencing sessions are performed. These positive outcomes are supported by various telehealth studies where videoconferencing has been used to monitor treatments undertaken remotely in a patient’s home [59, 60]. However, in contrast to monitoring with real-time videoconferencing, we note that compliance with treatment protocols did not appear to reflect any particular strategy employed to prepare the participant for the in-home tES and deliver the program such as training participants or caregivers in the use of in-home tES, or the use of customised headbands. However, this in no way should undermine the importance of training sessions or additional approaches to prepare participants for performing in-home tES. Indeed, there is some indication that use of multiple strategies (at least three different approaches) to prepare the participant and deliver the in-home tES program may be associated with greater protocol compliance. However, this requires further investigation. Therefore, at this stage we suggest a multifaceted approach combining several of the identified strategies to prepare and train the participant to use tES in their own home along with real-time videoconferencing to monitor stimulation may be best. Future work should continue to explore approaches to ensure delivery of in-home tES is an acceptable, easy and safe.

Aside from videoconferencing, there were several other methods that have been used to monitor in-home tES treatments. For example, participants were required to self-report treatments using either treatment diaries [44–46] or online tracking systems [39]. While self-reported approaches do provide a record of in-home tES use, the accuracy of this approach relies upon regular reporting and accurate recall. As self-reported approaches do not obtain real-time information relating to in-home tES sessions, research staff are unable to monitor treatment sessions. This may prevent the ability to ensure correct electrode preparation, set-up and starting of the stimulator. These difficulties faced during the treatment phase by the study participants might contribute to the lower compliance. One interesting observation is that the use of real-time videoconferencing for monitoring in-home tES may have to be ongoing, and regular, to help achieve best protocol compliance. This observation is supported by CK Loo, A Alonzo and J Fong [49], where compliance was reported as 90% with a protocol which included a videoconference performed for the initial in-home tES session only, with the remaining sessions monitored through phone and email contact.

There is also some indication that regular videoconferencing sessions may contribute to greater patient satisfaction with using in-home tES. Few studies, which did use videoconferencing to monitor in-home tES, reported that study participants were able to set-up the stimulator and begin treatment without difficulty and were interested in continuing treatment with in-home tES [39, 48, 50, 55, 56]. However, patient perspectives were not commonly reported by the studies included in this review and this is an area for future investigation. Ensuring higher levels of satisfaction may assist with improving compliance with home treatment programs and may help facilitates clinical translation of this approach.

While a concern regarding in-home tES may be greater risk of injury from stimulation, results from this review suggest that no severe adverse events associated with receiving stimulation occurred during in-home tES and that any reported adverse events were akin to those reported for tES trials performed in clinics or research facilities [26, 61]. Common adverse events that were reported included tingling, burning and itching sensations especially at the electrode placement site. One study did report that participants experienced poor sleep and mood changes [46]. It is not clear why these adverse events occurred in this study involving stimulation applied to either the auditory cortex or frontal cortex in people with tinnitus. However, we do note that this study used a passive monitoring approach of self-reported treatment diaries. This may have limited the ability of the research team to track the progression of these symptoms at each session and discuss approaches to manage, or prevent, their occurrence. Furthermore, we note that one seizure was reported but was unlikely related to the home tES as it occurred to a participant receiving sham stimulation who had a history of epilepsy. While there does not appear to be a relationship between monitoring strategies for in-home tES and the occurrence of adverse events, we suggest that real-time monitoring approaches, such as videoconferencing, should be used to ensure patient safety and limit potential for adverse events to occur.

A limitation of the current study is that it does not account for the various patient groups that were included. It is likely that different pathology and patient characteristics could influence treatment compliance and satisfaction with in-home tES. As the current literature around in-home tES continues to grow, it is recommended future studies investigate how different patient groups respond and accept in-home tES. It may be that specific monitoring approaches or strategies to implement in-home tES are required for different patient populations to ensure high levels of treatment compliance and satisfaction are achieved.

## Conclusion

Although in-home tES is a relatively new area of research, current evidence indicates that this is a feasible, acceptable and safe approach to deliver non-invasive brain stimulation treatment for a range of neurological and psychiatric conditions. This review indicates that the use of videoconferencing to monitor in-home tES can result in excellent levels of treatment fidelity and potentially greater participant satisfaction. While there are different approaches to implement and monitor in-home tES, we suggest real-time monitoring through videoconferencing should be included as one of these strategies in future studies. The area of in-home tES research is rapidly developing, driven in part by the ability to deliver consecutive stimulation sessions without overburdening people who are unwell by requiring that they travel frequently to receive treatment. We suggest future studies continue to explore patient groups which may benefit from this treatment approach and continue to monitor critical aspects of protocol compliance, adverse events and participant satisfaction. This information will help shape delivery of in-home tES to ensure that best possible services are provided.

## Abbreviations

PRISMA: Preferred reporting items for systematic reviews and meta-analysis
tACS: Transcranial alternating current stimulation
tDCS: Transcranial direct current stimulation
tES: Transcranial electrical stimulation
tRNS: Transcranial random noise stimulation
